# Urinary Proteome Analysis Identified Neprilysin and VCAM as Proteins Involved in Diabetic Nephropathy

**DOI:** 10.1155/2018/6165303

**Published:** 2018-04-29

**Authors:** Elena Guillén-Gómez, Beatriz Bardají-de-Quixano, Sílvia Ferrer, Carlos Brotons, Mark A. Knepper, Montserrat Carrascal, Joaquin Abian, José M. Mas, Francesca Calero, José A. Ballarín, Patricia Fernández-Llama

**Affiliations:** ^1^Molecular Biology Laboratory, Fundació Puigvert, Barcelona, Spain; ^2^Universitat Autònoma de Barcelona, Institut d'Investigació Biomèdica Sant Pau (IIB Sant Pau), Barcelona, Spain; ^3^Renal Transplantation Unit, Nephrology Department, Fundació Puigvert, Barcelona, Spain; ^4^Maragall Primary Health Care Center, Barcelona, Spain; ^5^Sardenya Primary Health Care Center, Barcelona, Spain; ^6^Epithelial Systems Biology Laboratory, National Heart, Lung, and Blood Institute (NIH), Bethesda, MD, USA; ^7^Proteomics Laboratory CSIC/UAB, Institut d'Investigacions Biomèdiques de Barcelona (IIBB-CSIC-IDIBAPS), Barcelona, Spain; ^8^Anaxomics Biotech SL, Barcelona, Spain; ^9^Hypertension Unit, Nephrology Department, Fundació Puigvert, Barcelona, Spain

## Abstract

Urinary proteome was analyzed and quantified by tandem mass tag (TMT) labeling followed by bioinformatics analysis to study diabetic nephropathy (DN) pathophysiology and to identify biomarkers of a clinical outcome. We included type 2 diabetic normotensive non-obese males with (*n* = 9) and without (*n* = 11) incipient DN (microalbuminuria). Sample collection included blood and urine at baseline (control and DN basal) and, in DN patients, after 3 months of losartan treatment (DN treated). Urinary proteome analysis identified 166 differentially abundant proteins between controls and DN patients, 27 comparing DN-treated and DN-basal patients, and 182 between DN-treated patients and controls. The mathematical modeling analysis predicted 80 key proteins involved in DN pathophysiology and 15 in losartan effect, a total of 95 proteins. Out of these 95, 7 are involved in both processes. VCAM-1 and neprilysin stand out of these 7 for being differentially expressed in the urinary proteome. We observed an increase of VCAM-1 urine levels in DN-basal patients compared to diabetic controls and an increase of urinary neprilysin in DN-treated patients with persistent albuminuria; the latter was confirmed by ELISA. Our results point to neprilysin and VCAM-1 as potential candidates in DN pathology and treatment.

## 1. Introduction

Diabetic nephropathy (DN) is the leading cause of end stage renal disease (ESRD) [1]. Incipient DN is characterized by the appearance of microalbuminuria that increases as the disease progresses and may lead to macroalbuminuria and renal failure. It is known that renin-angiotensin system (RAS) blockers, particularly angiotensin II (Ang II) antagonists such as losartan, can slow down the progression of ESRD [2].

Urine proteomics consists of a large-scale study in a single analysis to identify thousands of proteins and peptides. Urine proteomic investigations in DN identified potential biomarkers allowing an early detection of DN as well as prediction of normoalbuminuric diabetic patients prone to develop DN [3, 4]. Zürbig et al. also demonstrated the predictive value of urine proteomics for detection of progression to macroalbuminura [5]. Besides, the usefulness of urine proteomics to reveal potential biomarkers was evidenced by a multiple proteomic comparison researches in which several proteins differently abundant in patients with DN were identified. This was an important step forward to improve accurate diagnosis and understanding of the disease mechanisms [6, 7].

Despite this new progress, there is not yet an appropriate therapy to prevent DN. Moreover, it is common to find other clinical factors such as overweight, dyslipidemia, and hypertension in DN patients contributing to renal damage. In this work, we have studied incipient DN male patients before and after losartan treatment, and, in contrast with other studies, we have selected non-obese patients with a good blood pressure and lipid control, with the aim of improving the identification of factors closely related to the pathogenesis of DN.

## 2. Subjects and Methods

### 2.1. Patients

Twenty-one type 2 diabetic patients were enrolled in the study: 12 without DN (control patients) and 9 with incipient DN (DN basal). The inclusion criteria were (1) males ≥ 35 years old; (2) controlled hypertension by 24 h ambulatory blood pressure monitoring (ABPM) (24 h systolic BP (SBP) < 130 mmHg and 24 h diastolic BP (DBP) < 80 mmHg) [8]; and (3) absence (control cohort) or presence (DN patients) of persistent microalbuminuria: albumin/creatinine ratio from 2.26 mg/mmol to 22.6 mg/mmol at least in two out of three urine morning samples. The exclusion criteria were (1) estimated glomerular filtration rate (CKD-EPI) < 60 ml/min/1.73 m^2^; (2) having taken a RAS inhibitor ≤ six months prior to inclusion; (3) any cardiovascular event during the past year; (4) serum potassium > 5.0 mmol/l; (5) serum LDL cholesterol > 3.0 mmol/l, triglyceride > 1.7 mmol/l, or glycated hemoglobin HbA1c > 7.5% (>58 mmol/mol); (6) body mass index (BMI) ≥ 35 kg/mm^2^; (7) documented renal artery stenosis; (8) any condition that may alter the absorption, distribution, metabolism, or excretion of RAS inhibitor drugs; (9) history of malignancy in the last 5 years; (10) history or evidence of drug or alcohol abuse during the past year; (11) history of hypersensitivity to any RAS inhibitor; and (12) history of noncompliance with medical regimens.

The theoretical sample size necessary for obtaining statistically significant differences was calculated based on the standard deviation of the inclusion/exclusion criteria analytical parameters. Given the homogeneity of the subjects and the specificity of inclusion/exclusion criteria that made it difficult to find patients matching them, sample size was established at the lower limit of the interval obtained through the equation.

### 2.2. Blood Pressure, Blood, and Urine Analysis

SBP, DBP, mean BP (MBP), and heart rate over day, night, and 24 h were measured by 24 h ABPM (Diasys Integra II; Novacor, Paris, France). Office BP was recorded following the European guidelines [9].

Serum electrolytes (sodium and potassium) were analyzed by selective ion electrodes; serum urea, creatinine, lipidic profile, glycemic profile, and urine albumin and creatinine levels were determined by kinetic, colorimetric, or immunoturbidimetric assays, in the Roche Cobas® 6000 analyzer, following the manufacturer's instructions.

### 2.3. Urine Collection

First morning urine void was collected, centrifuged, and stored at −80°C until proteomic analysis.

### 2.4. Quantitative Liquid Chromatography Tandem Mass Spectrometry (LC-MSn) Analysis

Four biological replicates from each condition (control, basal, and treated) were processed. Each replicate was a pool of three or four different patients as appropriate.

#### 2.4.1. Sample Preparation

Samples (14–28 ml) were thawed and centrifuged (4000*g*, 4°C, 20 min), filtered (Steriflip PVDF 0.22 *μ*m pore, Millipore, Watford, UK), and concentrated using Amicon® centrifugal filters (Amicon Ultra 15 ml, Millipore) up to about 250 *μ*l final volume. Samples were evaporated in a SpeedVac (Thermo Electron, Waltham, MA, USA) to a final volume of about 50 *μ*l and albumin/IgG depleted using the PROTIA Proteoprep Immunoaffinity kit (Sigma-Aldrich, St Louis, MO, USA).

Each sample was immunodepleted separately. Pools of 3-4 different patients were prepared for further processing combining volumes of eluate containing equimolar amounts of protein (26.7 *μ*g each). A total of 12 pools were prepared (4 controls, 4 diabetic basal, and 4 diabetic treated).

#### 2.4.2. Protein Digestion and Peptide Labeling

Pooled immunodepleted samples (80 *μ*g of protein) were digested as previously described. Each tryptic peptide mixture was labeled with the corresponding tandem mass tags (TMT) (Thermo Fisher Scientific, Rockford, IL, USA). The TMT labeling kits used provide 6 different molecular labels. Thus, only 6 samples can be analyzed together per LC-MS experiment. So, the 12 pools available were analyzed in two independent experiments each containing 2 replicates (pools) of each class. In each experiment, the six differentially TMT-labeled samples were combined in a low-bind 1.5 mL Eppendorf tube, evaporated, and desalted using a C18 SPE cartridge (3 mL, 15 mg, Agilent Technologies, Waldbronn, Germany). The SPE eluates were evaporated and resuspended in 200 *μ*l of 30% ACN (0.1% formic acid).

#### 2.4.3. Sample Fractionation by Strong Cation Exchange Chromatography

SCX fractionation of the TMT-labelled peptide mixtures was performed on an Agilent 1100 HPLC system (Agilent Technologies) using a Polysulfoethyl A™, 50 × 2.1 mm, 5 *μ*m, and 200 Å column at a flow rate of 200 *μ*l/min. A linear NH_4_Cl gradient from 0 to 25% B in 38 min and then to 100% B in 20 min was used (buffer A: 30% ACN, 0.1% formic acid; buffer B: 30% ACN, 0.1% formic acid, 0.5 M NH_4_Cl). Six fractions were collected from minute 10 to 52.

#### 2.4.4. LC-MSn Analysis

One-fifth of each collected SCX fraction was analyzed by LC-MSn using an Agilent 1200 nano pump (Agilent Technologies) connected to a LTQ-Orbitrap XL instrument (Thermo Fisher Scientific) equipped with a nanoESI source (Proxeon, Odense, Denmark). Separations were carried out using a C18 preconcentration cartridge (Agilent Technologies) connected to a 15 cm-long 100 *μ*m i.d. C18 column (Nikkyo Technos Co., Japan). Separations were done at 0.4 *μ*l/min using a linear ACN gradient from 0 to 40% in 120 min (solvent A: 0.1% formic acid, solvent B: ACN 0.1% formic acid). The spectrometric analysis was performed in an automatic data-dependent mode. A full scan followed by 1 HCD and 1 CID MS/MS scans for the 5 most abundant signals was acquired (dynamic exclusion: 1, time window: 30 s).

#### 2.4.5. Database Search

Thermo RAW files were processed using the EasierMgf software. Database search was done using Proteome Discoverer v1.4 (Thermo-Instruments) with a 1% false discovery rate (FDR) and the UniProt 2014-10 database restricted to *Homo sapiens*. Search parameters were parent tolerance, 20 ppm; fragment tolerance, 0.8 Da; enzyme, trypsin; missed cleavages, 1; fixed modifications, TMTsixplex (N-terminal, K), carbamidomethyl (C); and variable modifications, oxidation (M).

### 2.5. Bioinformatic Analysis of Proteomic Data

Based on artificial intelligence and pattern recognition techniques, Therapeutic Performance Mapping System (TPMS, Anaxomics Biotech) [10, 11] creates mathematical models that integrate all the available biological, pharmacological, and medical knowledge to simulate human physiology in silico. TPMS technology includes two different and complementary strategies to solve mathematical models:

- Artificial neural networks (ANNs): this strategy is able to identify relationships among regions of the network (generalization). These provide a predictive value that infers the probability of the existence of a specific relationship between two or more sets of proteins (in this case, each protein and DN), based on a validation of the predictive capacity of the model towards what is described in databases.
- Sampling methods: this second strategy allows to trace back observed effects to molecules and is normally applied once a key region of the map has been identified (using an ANN or as suggested by experimental work). Once a response is identified to a specific stimulus (e.g., a drug target, as identified with an ANN), it is possible to analyze mechanisms of action using the sampling methods strategy.

Mathematical models are able to suggest mechanistic hypotheses that are consistent with actual biological processes. Finally, the comparative analysis of healthy and DN mathematical models revealed functional properties and mechanistic insights specific of the pathological state of interest.

**Figure 1:**
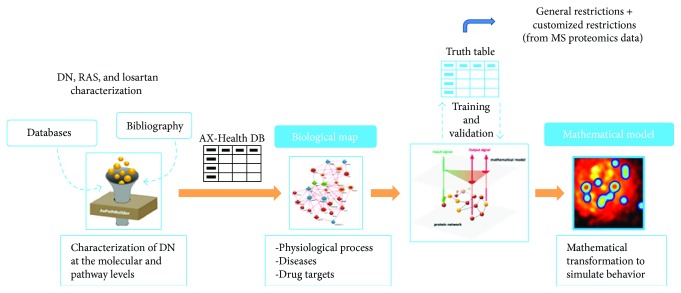
Mathematical model generation process. It includes the creation of a Biological Effector Database (BED), the construction of a biological map, and, finally, the achievement of the mathematical model.

The process comprises four steps (Figure 1): (1) collection of scientific knowledge based on hand-curated databases that relate biological processes to their molecular effectors (BED) (including a specific DN, type 2 diabetes, and RAS pathway characterizations); (2) preparation of a human biological network focused on DN based on data retrieved from both public and private external databases such as KEGG, BioGRID, IntAct, REACTOME, and MINT; (3) subsequent generation of mathematical model, whereby the biological map is transformed into a mathematical model capable of both reproducing existing knowledge and predicting new data. To do this, the mathematical models were previously trained using a collection of known input-output physiological signals (e.g., a drug indication relationship), these being obtained from the literature mining and a compendium of databases that accumulate biological and clinical data [12–17], namely:

- Model inputs: for example, information about drugs provided by DrugBank [18], since they inhibit or activate one or more nodes of the model (their targets) triggering a perturbation through the system,
- Model outputs: for example, experimental microarray data (upregulated or downregulated proteins, after the treatment). The collection of known input-output physiological signals generates a list of physiological rules or principles found to apply to all humans, or to particular pathophysiological conditions, that act as mathematical restrictions. These sets of rules are collected to form a *Truth Table*, a collection of mathematical restrictions that include the available biological knowledge on the constructed networks, together with knowledge derived from DrugBank, and the statistically significant differentially expressed proteins from transcriptomic data. Furthermore, models able to simulate the physiological behavior of diabetic patients suffering DN were generated including the differentially expressed proteins extracted from the different group comparison,

and (4) extraction of biological and clinical conclusions. To extract conclusions, the pairs of mathematical models of interest were compared with the aim of identifying the key proteins involved in the pathophysiology of the DN and in the efficacy of losartan:

- Identification of the key proteins and mechanism of action associated with DN pathophysiology:
  - Model of diabetic patient suffering DN versus healthy model
- Identification of the key proteins and mechanism of action associated with losartan effect:
  - Model of diabetic patients suffering DN and treated with losartan versus model of diabetic patients suffering DN
  - Model of diabetic patients suffering DN and treated with losartan versus model of diabetic patients

Sampling methods were used to describe with high capability all plausible relationships between nodes of the mathematical models. As the number of restrictions is always smaller than the number of parameters required by the algorithm, any process modeled by TPMS considers a population of different solutions, currently set around 10^6^–10^9^ since this interval is estimated to faithfully portray nature. Subsequently, TPMS traces the most probable pathways (in biological and mathematical terms) among all the possible pathways leading from the stimulus to the response through the biological network.

Mathematical models should be able to weigh the relative value of each protein ratio (node). In the mathematical algorithm, each parameter corresponds to the relative weight of a link connecting nodes (genes/proteins) in a graph (protein map). Using the sampling methods, we generated populations of solutions that comply with the biological restrictions of the Truth Table. This approach allows tracing back the biological effects on molecules or triggering effectors by analyzing the different populations of solutions. Thus, the population of solutions accounts for the variety of physiological responses that may occur in human populations. The mathematical model is challenged with the stimulus and the response, and we traced the most probable path (in biological and mathematical terms) that leads from the stimulus to the response through the biological network. Thus, it identifies the most probable MoA that achieves a physiological response when the system is stimulated. For the analysis, we used those solutions that comply with the general knowledge collated in the Truth Table (high accuracy values). That is, only MoAs that are plausible from the standpoint of currently accepted scientific understandings and the restrictions applied were considered in the analysis.

### 2.6. Urinary Neprilysin and VCAM-1 ELISA

Their abundance was tested in urinary samples from patients and controls using a specific enzyme-linked immunosorbent assay (ELISA). Urine neprilysin (MyBioSource, San Diego, California, USA) and human vascular cell adhesion molecule-1 (VCAM-1) ELISA (Sigma-Aldrich, St. Louis, Missouri, USA) were performed following the manufacturer's instructions.

### 2.7. Presentation of Data and Statistical Analyses

Patient clinical data: quantitative data are presented as the mean ± standard deviation (SD). Statistical comparisons were performed by *t*-test (unpaired or paired as appropriate using SPSS 17.0).

Proteomic data analysis: DanteR [19] was used for relative quantification. Only unique peptides were considered for the analysis. Tandem mass tag (TMT) reporter intensity data were normalized using the Loess method followed by adjustment with central tendency. ANOVA was performed and adjusted by false discovery rate (FDR) correction. Two different comparisons were carried out: (1) basal and treated versus control and (2) treated versus basal. Differential proteins were selected using an adjusted *p* value cutoff of 0.05 and a ratio < 0.7 (down) or >1.3 (up).

Bioinformatic analysis: mathematical models allowed the identification of key proteins associated with DN pathology and treatment efficacy; *p*-values used to select these key proteins were ≤0.005 and ≤0.05, respectively.

## 3. Results

### 3.1. Patients

**Figure 2:**
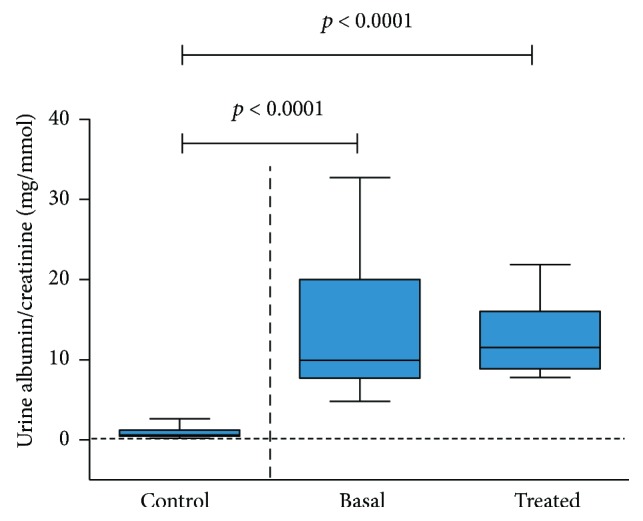
Box-and-whisker plots of urine albumin/creatinine ratios in mg/mmol in the three studied groups. Each value was calculated from three successive measurements. *N* control = 11, *N* basal = 9, and *N* treated = 9; statistical test: Student's *t*-test.

Clinical and demographic characteristics of the study population are shown in Table 1. All patients included in the study were over 60 years old. Control and DN basal groups were balanced regarding baseline characteristics except for albuminuria levels. None of the patients had diabetic retinopathy. The 9 DN patients were treated with losartan during three months. After this period, there were no differences in terms of albuminuria, HbA1c, eGFR, cholesterol, and triglycerides levels.  Figure 2 shows albumin/creatinine ratio in the three groups studied. Losartan treatment in DN patients maintained office BP (SBP 144.0 ± 17.7 versus 140.0 ± 24.8 mmHg; DBP 73.6 ± 6.6 versus 75.3 ± 6.8 mmHg, basal versus treatment, resp.). Antidiabetic drugs are specified in Table 1. Ten patients from the control group and 4 from the DN group took statins. Five patients from the control group and 4 from the DN group took antiplatelet agents, being aspirin the most used.

### 3.2. Protein Identification and Relative Quantification

10780 spectra corresponding to 2520 nonredundant peptides were identified through database search (1% FDR). For quantitative analysis, only peptides identified as unique (i.e., peptide sequences belonging to one single protein in the database) were considered. Overall, a total of 688 proteins could be quantified from 2191 nonredundant unique peptides. The mass spectrometry proteomic data have been deposited to the ProteomeXchange consortium via the PRIDE [20] partner repository with the dataset identifier PXD009303. This information is also available in the Supplementary data (available here).

### 3.3. Proteomic Data Processing and Enrichment Analysis of Differentially Abundant Proteins

**Figure 3:**
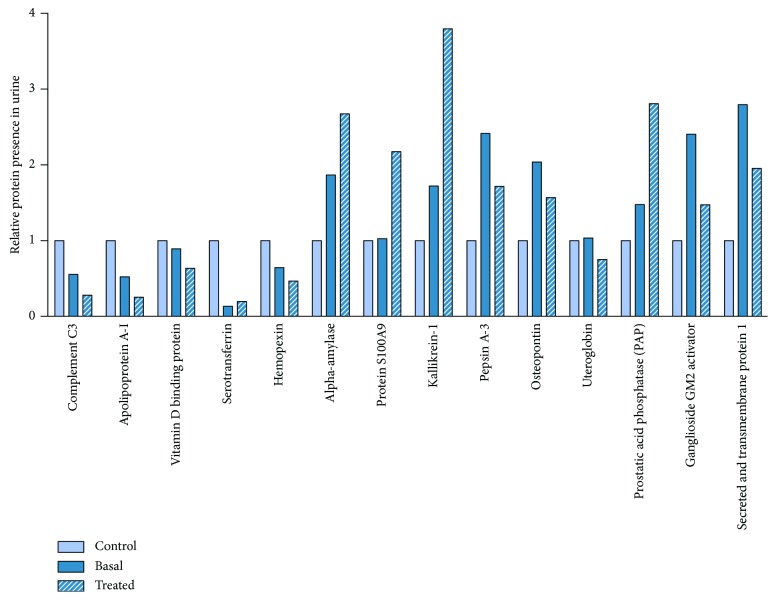
Differentially abundant proteins among the three cohorts. Data presented as a relative change from the control cohort.

We identified 166 differentially abundant proteins in basal versus control comparison, 27 between treated and basal cohorts, and 182 in treated versus control comparison (Table 2). Detailed information is provided in the supplementary data. We predicted 138 and 13 proteins as direct effectors of DN and RAS efficacy, respectively, according to its molecular characterization in the Biological Effector Database (BED) [21]. Furthermore, we found 14 differential proteins common to the three evaluated cohorts considering both upregulated and downregulated proteins. One of them, osteopontin, a bone matrix protein and proinflammatory cytokine, was also predicted as a direct effector of DN according to its BED molecular characterization (Figure 3).

The enrichment analysis of the differential proteins between basal and control cohorts revealed 344 enriched pathways, 263 between treated and basal cohorts, and 352 between treated and control cohorts. Interestingly, some of them are related to DN and RAS according to an artificial neural network (ANN) [22] analysis. This analysis identifies associations among different regions of the network, such as potential relationships between the enriched protein sets and DN and RAS. Specifically, the vasoactive hormone pathway is enriched in the treated versus basal cohorts' comparison. The inflammation associated with DN is an enriched pathway in the comparisons between treated versus basal and basal versus control cohorts.

### 3.4. Clustering Analysis

**Figure 4:**
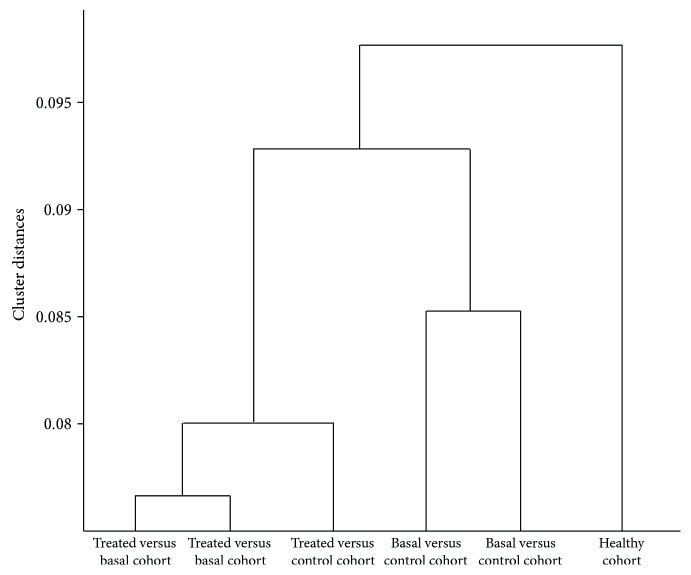
Hierarchical clustering of the mathematical models. The dendrogram was build using the Hausdorff distance between the set of mechanisms of action defined by the models in duplicate.

Results of hierarchical clustering, according to mathematical models, are represented in Figure 4 as a dendrogram to show distances in terms of protein expression between groups.

### 3.5. Key Proteins Involved in DN and RAS Efficacy

Comparative analyses among the generated mathematical models predicted 80 proteins with an important role in DN (key proteins) (supplementary data): (1) 14 of them are measurable in urine; (2) 20 have been previously considered effectors of DN; (3) 2 have also been identified as differentially abundant proteins from the cohort comparisons of proteomic data; and (4) 45 of the key proteins are directly linked to some of the differentially present proteins identified from the cohort comparisons of proteomic data.

Regarding losartan effect, comparative analysis from mathematical models predicted 15 key proteins (supplementary data): (1) 7 are measurable in urine; (2) none are RAS efficacy key proteins according to the molecular characterization in our BED; (3) 4 have been also identified in the differential presence analysis of proteomic data; and (4) 9 are directly linked to one or several of the differentially present proteins identified from the cohort comparisons of proteomic data.

An effector protein is defined as an essential protein in the disease pathology according to its molecular characterization in BED and published literature, whereas a key protein is predicted through the analysis of mathematical models. Key proteins can also be effector proteins or new potential candidates (in the disease pathology or in the treatment efficacy), the role of which has not been described before. In this work, we detected 5 DN effector proteins differentially abundant among the three cohorts: osteopontin, neprilysin, fibronectin, kininogen-1, and VCAM-1 (Table 3(a)). Neprilysin and VCAM-1, however, are the only ones that are also DN disease key proteins. Additionally, we predicted 4 losartan effect key proteins differentially abundant among the three cohorts: neprilysin, VCAM-1, kininogen-1, and alpha-2-macroglobulin. Only alpha-2-macroglobulin is not a losartan effector protein (Table 3(b)).

Finally, we predicted 7 key proteins in both DN pathophysiology and losartan effect (Table 4). VCAM-1 and neprilysin stand out from the others because they are differentially abundant in the urine proteome.

### 3.6. Urinary Neprilysin and VCAM-1 ELISA

**Figure 5:**
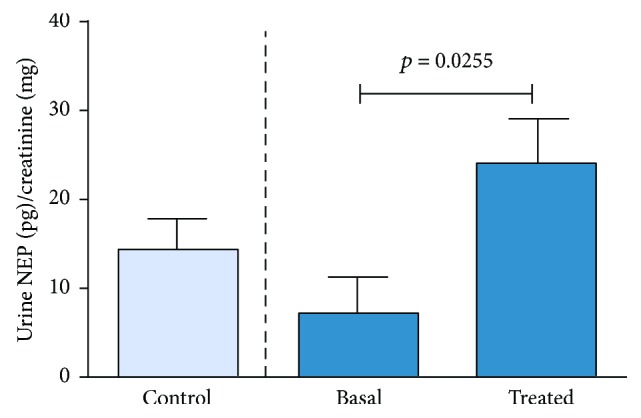
Quantification of urinary neprilysin (NEP) by ELISA showed an increased presence of this protein in losartan-treated patients compared to basal cohort. *N* control = 11, *N* basal = 9, and *N* treated = 9; statistical test: Student's *t*-test.

We were only able to detect VCAM-1 in one of the urine samples among the three cohorts through ELISA analysis. Regarding neprilysin, urine levels were higher in DN losartan-treated patients than in the untreated patients (*p* = 0.0255) (Figure 5), reinforcing the results obtained in the proteomic analysis.

## 4. Discussion

Albuminuria is not specific for DN and is highly variable [23]. In order to identify alternative biomarkers, we performed urinary proteomic analysis in diabetic and incipient DN patients, the latter before and after losartan treatment. Several publications support that profiling of the urinary proteome can be useful to diagnose DN and identify novel biomarkers [6, 7, 24, 25]. In our study, patients' selection criteria were strict, avoiding confounder factors such obesity, uncontrolled BP, or dyslipidemia, which are commonly associated comorbidities that also induce albuminuria. Therefore, differences in urinary proteome would correspond to the disease itself and not to associated secondary problems.

In contrast to other proteomic studies, our results were further analyzed by bioinformatic tools including mathematical models and several databases in order to reach more specific and distilled information. Thus, we predicted 5 proteins known to be involved in DN pathophysiology and 4 associated with losartan treatment. Two of them, VCAM-1 and neprilysin, are effector proteins of both disease and treatment efficacy, making them the most relevant proteins at this early stage of the disease. Levels of VCAM-1, a candidate biomarker of renal pathology [26], correlate with albuminuria in diabetic hypertensive patients [27] and with the number of infiltrating immune cells [28]. Its expression is increased in the kidneys from DN patients [29], probably because Ang II upregulates VCAM-1 [30]. In accordance with these results, we observed that VCAM-1 urine levels were increased in basal albuminuric DN patients compared to diabetic controls without renal damage, supporting its role in this process. In fact, there are preclinical studies describing the blockade of adhesion molecules as a potential therapeutic target [31].

Urine proteome also showed changes in neprilysin abundance that were also confirmed by ELISA. Neprilysin is a metalloprotease that inactivates several peptides including natriuretic peptides, bradykinin, and endothelins. It is particularly abundant in the kidney where it is bound to plasma membrane, but it is also present in a soluble form in urine and blood. The urine form appears to reflect the activity of the enzyme in the kidney [32, 33]. Our proteomic results indicate an increase of urinary neprilysin after losartan treatment in DN patients showing persistent albuminuria. To our knowledge, this is the first study that observes changes in urine neprilysin after losartan treatment in incipient DN patients. The increase of Ang II, by losartan, may favor the activity and/or expression of neprilysin through the alternative RAS activation towards the formation of Ang (1–7), without neglecting the contribution of angiotensin-converting enzyme 2 (ACE2). Indeed, the selective neprilysin inhibitor SCH39370 abolished the formation of Ang (1–7) [34].

Several studies demonstrate a potential role of neprilysin in renal damage [35]. A DN animal model showed greater attenuation of albuminuria when treated with a neprilysin inhibitor compared to a RAS blocker [36]. Vasodilatation is associated with natriuretic peptides and may result in reductions of intraglomerular pressure and proteinuria [37, 38]. Neprilysin inhibition could increase these effects and can also impair breakdown of Ang II. Beneficial effects of neprilysin inhibition are enhanced when combined with a RAS inhibitor, which has led to the development of dual inhibition. Additionally, there is an ongoing clinical trial testing the nephroprotection effects of this double inhibition [35, 39].

Neprilysin metabolizes Ang I to Ang (1–7) and inactivates bradykinin, whereas angiotensin I-converting enzyme (ACE) catalyzes the conversion of Ang I to Ang II and it is able to inactivate bradykinin. Accordingly, changes in RAS are accompanied by changes in kallikrein-kinin system, seeming necessary to control both systems in the treatment and monitoring of DN [40]. Our results also show differences regarding kallikrein-kinin system proteins such as urine kininogen-1 and kallikrein-1. These proteins should be studied in a larger cohort to confirm their role in this disease and in RAS system pathway.

Proteomic results also show differences in extracellular matrix proteins such as fibronectin and osteopontin. Osteopontin is a glycoprotein that blocks apoptosis of macrophages and T-cells as well as fibroblasts and endothelial cells exposed to harmful stimuli. It has been suggested to have a role in albuminuria and mesangial expansion in DN [41]. There are contradictory results regarding urine osteopontin as a DN progression biomarker [42, 43]. Our results show increased levels of osteopontin in DN patients compared to diabetic controls. These results are in accordance to previous studies that demonstrated that increased Ang II stimulates the production of osteopontin in the glomerulus in DN [44]. Nevertheless, DN patients treated with losartan show lower osteopontin than basal without changes in albumin excretion. This may be explained because Ang II mediates osteopontin synthesis [45, 46] and blockade of AT1 receptors could prevent its effects.

Fibronectin is another extracellular matrix glycoprotein important in fibrosis. Our results show increased levels of fibronectin in DN patients compared to diabetic controls. Murine diabetic models develop nephropathy and increase glomerular expression and accumulation of type IV collagen and fibronectin [47–50]. Furthermore, AT1 receptor blocking by losartan did not influence urinary fibronectin [51, 52]. Our results confirm that high urinary fibronectin in DN patients is not affected by losartan treatment, suggesting an AT1 receptor-independent pathway.

Progression of DN includes inflammation of glomeruli and tubulointerstitial regions accompanied by expression of adhesion molecules and chemokines, resulting in macrophage infiltration into renal tissues [53]. Vasoactive hormones are known to be key mediators of renal injury [54]. Our urine proteomic results show proteins related to inflammation and vasoactive pathways, such as osteopontin, VCAM, neprylisin, and kininogen, that are differentially expressed in the cohorts studied. Although our patients are in an initial stage of the disease, the enrichment results already show alterations of these pathways, suggesting an early involvement in incipient DN.

In our DN patients, factors associated with albuminuria are closely controlled and this could explain partially the lack of clinic response to losartan treatment. Although activation of RAS is related to albuminuria, there are other critical factors associated with its development that probably will be more relevant in non-obese DN patients with good blood pressure, lipid, and glycemic control. There are also other reasons that could explain the lack of therapeutic responses to RAS inhibition including incomplete inhibition of RAS, lack of effects on structural hallmarks of DN, and reversibility of established renal lesions on these patients [55, 56]. Evidences suggested that microalbuminuria may not be the ideal marker of DN progression [57, 58]. Moreover, our proteomic approach pointed out some possible candidates involved in DN and losartan treatment since differences were statistically significant among the three studied groups. A validation study, on a greater cohort and during a longer period, would be necessary.

In conclusion, our results point to neprilysin and VCAM-1 as possible candidates involved in incipient DN pathology and RAS inhibition treatment in elderly males. If ongoing clinical trials with double inhibition of RAS and neprilysin are satisfactory, they could help in the clinical management of the disease. Moreover, urine detection of these proteins could serve as potential new tools as DN progression biomarkers.

## Figures and Tables

**Table 1:** Clinical and demographic characteristics of the study population.

	Controls	DN basal	DN treated	Statistics (student *t*-test)
*N* (gender)	12 (males)	9 (males)		N/A
Nonsmokers/smokers/ex-smokers	4/2/6	0/5/4		N/A
Age (years)	69.6 ± 9.6	71.0 ± 7.0		0.714
BMI	28.1 ± 5.2	27.0 ± 2.3		0.572
Time of type 2 DM (years)	13.8 ± 7.9	12.2 ± 4.5		0.591
HbA1c (%) (mmol/mol)	6.8 ± 0.8 (51 ± 9.0)	7.0 ± 0.7 (53 ± 7.6)	7.0 ± 0.7 (52.4 ± 7.4)	0.467
Cholesterol total (mmol/l)	4.1 ± 0.7	4.2 ± 0.8	4.3 ± 0.6	0.773
Cholesterol LDL (mmol/l)	2.1 ± 0.6	2.1 ± 0.6	2.3 ± 0.5	0.884
Triglycerides (mmol/L)	1.1 ± 0.4	1.3 ± 0.4	1.7 ± 0.8	0.196
eGFR (CKD-EPI) (ml/min/1.73 m^2^)	80.4 ± 10.9	89.1 ± 16.8	85.4 ± 19.0	0.1662
Urine albumin/creatinine (mg/mmol)	0.68 ± 1.0	13.6 ± 12.2	12.5 ± 4.8	*p* < 0.0001
ABPM				
24 h SBP (mmHg)	119.4 ± 11.8	121.4 ± 11.4	n.d.	0.869
24 h DBP (mmHg)	70.8 ± 5.0	69.2 ± 7.5	n.d.	0.509
Antidiabetic drugs				
Insulin (*N*)	1	1		N/A
Metformin (*N*)	8	9		N/A
Sulfonylureas (*N*)	6	1		N/A
Other oral drugs (*N*)	2	1		N/A

Data are expressed as the mean ± SD; the statistics column refers to the comparison between control patients and DN patients before losartan treatment (basal). All comparisons between basal and treated DN patients were nonsignificant. ABPM: ambulatory blood pressure monitoring; BMI: body mass index; DM: diabetes mellitus; DN: diabetic nephropathy; DBP: diastolic blood pressure; eGFR: estimated glomerular filtration rate; HbA1c: glycated hemoglobin; n.d.: not determined; SD: standard deviation; SBP: systolic blood pressure; N/A: not applicable.

**Table 2:** Summary of differentially abundant proteins identified for each evaluated cohort comparison that are also direct effectors of DN according to its molecular characterization in the Biological Effector Database (BED).

	Number of differentially expressed proteins			Number of DN effectors		
	Total	Upregulated proteins	Downregulated proteins	Total	Upregulated proteins	Downregulated proteins
Basal versus control	166	23	143	3	0	3
Treated versus basal	27	14	13	2	1	1
Treated versus control	182	26	156	3	3	0

DN: diabetic nephropathy.

**(a):** 

Protein name	Basal^∗^ versus control	Treated^∗^ versus basal	Treated^∗^ versus control
Osteopontin	Downregulated	Upregulated	Downregulated
Neprilysin	—	Downregulated	—
Fibronectin	Downregulated	—	Downregulated
Kininogen-1	—	—	Downregulated
VCAM-1	Downregulated	—	—

**(b):** 

Protein name	Basal^∗^ versus control	Treated^∗^ versus basal	Treated^∗^ versus control
Neprilysin	—	Downregulated	—
VCAM-1	Downregulated	—	—
Kininogen-1	—	—	Downregulated
Alpha-2-M	—	—	Upregulated

^∗^Cohort used as reference group. VCAM-1: vascular cell adhesion protein 1; Alpha-2-M: alpha-2-macroglobulin; —: no changes observed.

**Table 4:** Summary of the key proteins in both DN pathophysiology and losartan effect. The table indicates whether (1) the protein is a DN effector and for positive instances the process with which the protein is associated, (2) the protein is also differentially abundant from the cohort comparison (*d* = 0) or it is directly linked to one of them (*d* = 1), and (3) if the protein is easily measurable in urine according to bibliography review.

Protein	DN effector		Presence in proteomic data			Urine presence
	Yes/no	Process	*d* = 0	Comparison	*d* = 1	Yes/no
Neprilysin	Yes	Vasoactive hormones	Yes	Treated versus basal	Yes	Yes
Kallikrein	No		No	NA	Yes	Yes
Angiopoietin-2	Yes	Activation of angiogenesis	No	NA	No	Yes
Signal transducer and activator of transcription1-alpha/beta	Yes	JAK/STAT pathway alterations	No	NA	No	No
MAD homolog 7	Yes	Inflammation	No	NA	Yes	Yes
VCAM-1	Yes	Inflammation	Yes	Basal versus control	No	Yes
NADPH oxidase	No		No	NA	Yes	No

DN: diabetic nephropathy; N/A: not applicable; MAD homolog 7: mothers against decapentaplegic homolog 7; VCAM-1: vascular cell adhesion protein 1; NADPH oxidase: nicotinamide adenine dinucleotide phosphate hydrogen oxidase; JAK/STAT: Janus kinase/signal transducer and activator of transcription.

## Supplementary Materials

Supplementary Materials Supplementary Tables: the excel file includes (1) peptides identified by urine proteomics with intensities and identification scores: all peptides and protein groups (tab “All_data”) and unambiguous peptides pointing to a single protein that were used for statistical analysis (tab “Unique”); (2) differentially expressed proteins identified for each evaluated cohort comparison and which are direct effectors of DN, as well as the DN motives associated; (3) DN (tab “Key proteins DN”) and losartan (tab “Key proteins losartan”) key protein data containing the following columns: activation (sign of activation); DN effector (indicates whether it is a DN effector and the associated motive); presence in cohort comparisons in our proteomic data (the protein is also differentially abundant from the cohort comparison (*d* = 0) or it is directly linked to one of them (*d* = 1); urine presence (if the protein is easily measurable in urine according to bibliography review). Data added as an additional file through Manuscript Tracking System.
